# Towards Positive Aging: Links between Forgiveness and Health

**DOI:** 10.21926/obm.geriatr.2002118

**Published:** 2020-05-12

**Authors:** Noah J. Webster, Kristine J. Ajrouch, Toni C. Antonucci

**Affiliations:** 1. Institute for Social Research, University of Michigan, Ann Arbor, Michigan, United States; 2. Department of Sociology, Anthropology, Criminology, Eastern Michigan University, Ypsilanti, Michigan, United States; 3. Department of Psychology, University of Michigan, Ann Arbor, Michigan, United States

**Keywords:** Forgiveness, physical health, interpersonal resources, transgressions

## Abstract

Forgiveness may serve as an essential positive resource to help individuals cope emotionally with stressful events, ultimately influencing health. Examination of how individuals forgive within the context of close relationships can provide useful information about positive aging. In this study, we examine how the severity of a recent transgression committed by a spouse/partner or other close social relationship is associated with self-reported physical health among older adults. We also examine how state forgiveness (i.e., in context of a specific event) can offset the potentially negative impact of transgressions on health and further compare the impact when the transgressor is a spouse/partner versus another close social relationship. Data are from the Detroit Community Survey, a cross-sectional survey of social relations, forgiveness, humility, and health in the Detroit Metropolitan Area. Respondents age 50 and older were selected for analysis (N=380). Structural equation models indicated that greater transgression severity was associated with worse self-rated health. Further, state forgiveness was found to play a significant moderating role. Among older adults who were more likely to forgive their transgressor, experiencing a more severe transgression was associated with worse health. In contrast, among older adults less likely to forgive, there was no association between transgression severity and self-rated health. Additionally, among older adults less likely to forgive, the transgressor being a close other social relationship was associated with worse health compared to when it was a spouse/partner. In contrast, when more likely to forgive there was no association between who the transgressor was and self-rated health. This study contributes to a better understanding of how interpersonal stress, specifically a recent transgression experienced within the context of close social relationships, can be harmful to older adults’ health. Findings highlight the importance of forgiveness as a resource that can help facilitate positive aging.

## 1. Introduction

Positive aging references the ability to thrive in later years despite challenges that accompany the process of growing older [1]. The temperance virtue of forgiveness merits attention because of its potential role in curbing the tendency to hate [2]. Forgiveness is defined as a prosocial response in which there is a change in one’s thoughts, emotions, and behaviors toward a blameworthy transgressor [3]. Forgiveness includes the absence of negative qualities (e.g., the desire to seek revenge or avoid the transgressor) as well as the presence of positive qualities (e.g., feelings of beneficence and good will) [4]. Examination of the role of forgiveness within the context of specific relationships can provide useful information about positive aging in that it may serve as a critical resource to help individuals cope emotionally with stressful and/or traumatizing life events, ultimately influencing health.

Forgiveness is especially relevant for understanding close interpersonal relationships. Given the importance of close social ties in facilitating good health in later life, interpersonal stress experienced among close ties may be especially harmful to older adults. One type of interpersonal stress that has received less attention in the literature is transgressions, i.e., when a person feels harmed by another. Previous studies have documented how the frequency of negative interactions and conflict more generally experienced within close social relationships are associated with negative health outcomes [5, 6]. Despite this growing literature and greater understanding of the impact of interpersonal stress on positive aging, little is known about how characteristics of these transgressions influence outcomes such as physical health. Further, little is known about how interpersonal resources can offset the negative impact of experiencing a transgression. This information is needed to design effective positive aging interventions. In the present study, we compare less severe transgressions (e.g., annoyances) to those that are more severe by examining the association between transgression severity and physical health among older adults. We also examine how state forgiveness, i.e. feelings of forgiveness towards a specific person and event, can offset the potentially negative impact of experiencing a recent transgression. Further, we compare the role of forgiveness within the context of different relationships, e.g., when the transgressor is a spouse/partner versus other close social relationship.

### 1.1 Theoretical Frameworks

#### 1.1.1 Positive Aging

Positive aging is a broad perspective that is informed by multiple theories and models of human development and aging. One such model is the successful aging model [7-9], which articulates multiple dimensions of what it means to successfully age. One dimension of successful aging is being in good health, specifically avoiding disease and disability and maintaining good physical and cognitive function. This model guides and informs our focus on physical health as an outcome of positive aging. Our study is also grounded in positive psychology, specifically, Seligman’s [10] PERMA model which argues that well-being is influenced by five factors, one of which is positive relationships. In this study we examine the role of state forgiveness as a resource that may help maintain positive close relationships essential for older adults’ physical well-being.

#### 1.1.2 Forgiveness

There is a large theoretical literature on the topic of forgiveness spanning different types of forgiveness, antecedents, and outcomes. Two perspectives most relevant for the present study are the interpersonal process of forgiveness and unforgiveness [11] and the stress-coping model of forgiveness [12,13]. We consider each next.

The interpersonal process of forgiveness and unforgiveness model aims to explain circumstances surrounding a negative relational event, such as a transgression. The model begins with the premise that forgiveness and unforgiveness are different. Unforgiveness involves “resentment, bitterness, and hatred” while forgiveness is an “internal choice (either unconscious or deliberate) to relinquish unforgiveness” ([11] p. 386). Worthington and Wade [11] argue that transgressions occur in the context of a relationship that is perceived prior to the event affectively in terms of being positive and negative. When a transgression occurs, it is first subjectively appraised, and then reacted to emotionally (i.e., emotional forgiveness) or behaviorally (i.e., decisional forgiveness) [14]. Decisional forgiveness is argued to be more similar to reconciliation and thus may impact health indirectly by promoting positive relationships [14]. In contrast, emotional forgiveness, focused on in this study, is expected to alter the impact of stress by reducing negative and promoting positive affect [14]. Therefore, emotional forgiveness may play a direct role in altering the stress-health link.

The stress-coping model of forgiveness [12, 13] is based on and extends Lazarus and Folkman’s more general stress-coping theory [15, 16] to incorporate interpersonal transgressions and forgiveness. The model starts from the premise that the experience of transgressions are stressful, which are then appraised, resulting in a stress response [17]. Forgiveness is viewed as one of many possible coping responses to the experience of stressful transgressions [12, 13] that may be beneficial for minimizing the impact of stress on physical health [14].

#### 1.1.3 Relationship Context

In the two aforementioned models, relationship context is conceptualized to play an important role in the forgiveness process. While forgiveness is viewed as an intra-individual response, it is situated within an interpersonal context [17, 18]. This suggests research is needed to compare the role that forgiveness plays in the face of transgressions encountered across different relationships (e.g., marital/partner v. other close relationships such as adult children, friends, etc.). In the case of ongoing committed relationships, the elimination of negative emotions, it is argued, may not be sufficient given the likelihood of frequent contact. As a result, the victim and transgressor may additionally seek a positive emotional balance [19]. McCullough’s [20, 21] valuable relationship hypothesis further articulates the important role of relationship context. The hypothesis suggests that the more a relationship is perceived to have long-term value, the more motivation there may be to return to pre-transgression levels of interaction.

### 1.2 Links between Transgressions and Health

Close interpersonal relationships are generally viewed through a positive lens as a resource that can protect older adults from loneliness and social isolation [22-24]. However, it is now also generally well known that these same close relationships can be a source of stress for older adults, with negative influences on health in later life [25-28].The experience of stress and the resulting impact on health outcomes is well documented [29, 30]. Further, the impact of stress on health is known to be even stronger in later life [31]. Studies have found that the mental health impact of stress experienced in the context of interpersonal ties is more pronounced and lasts longer than the impact of non-interpersonal stress [32, 33]. This highlights the important need for research to identify resources that can help older adults manage and neutralize the negative impact of these stressful interpersonal experiences. Doing so can help older adults maintain strong social ties and maximally benefit from the positive aspects associated with these ties.

Stress experienced in the context of social relationships (i.e., interpersonal stress) specifically has been linked to poor physical health outcomes. Mechanisms explaining this link include the experience of depression, inflammation [34], and increases in blood pressure [35], all of which are linked to physical health [30]. Also, when interpersonal stress or conflicts are left unresolved, social isolation may be an outcome [36], which is known to be detrimental to health [24, 37, 38]. The unique and independent role of negative versus positive interpersonal interactions has also been detailed by Rook [6, 39], who found that negative interactions have a more powerful effect on mood and psychological well-being. Despite a greater understanding of the negative influences of interpersonal stress on health in later life, few studies have examined how a range of transgressions (e.g., from minor annoyances to being harmed more seriously) in the context of close social relationships can impact health [5].

### 1.3 Interpersonal Stress in Specific Relationships

It has been argued that conflict in voluntary relationships (e.g., friends and romantic partners) can undermine the relationship, whereas conflict in obligatory (family) relationships may have little impact on the stability of the tie [5]. However, this may not hold true at all life stages. Spouses/partners play a particularly powerful role in influencing the health of older adults. For example, it is well documented that married people or those living with a partner generally report better health than their non-married/partnered counterparts [40-42]. Also, spouses/partners are overwhelmingly primary caregivers for older adults experiencing health declines. This highlights the important role of this close social tie for facilitating positive aging. Therefore, being hurt by this person may be especially harmful to health.

Previous research suggests negative aspects of martial relationships compared to friendship ties can have differential, in fact more severe, impacts on health [43]. Along these lines, Kiecolt- Glaser and colleagues [44] found that among couples, wound healing was slower after a hostile interaction than compared to an interaction in which the couple exchanged social support. Similarly, Birditt and colleagues [45] found links between higher levels of marital stress and larger waist circumference. Less is known though about the impact on health of interpersonal stress experienced in the context of other close relationships compared to spouses/partners.

### 1.4 Forgiveness

The ability to forgive has been found to increase as people get older [3, 46, 47]. Further, the link between forgiveness and health has been found to be stronger among middle aged and older adults compared to younger adults [46]. Multiple studies have found that more forgiveness benefits multiple indicators of physical health [48] including self-rated health [46, 49-51], lower blood pressure [50, 52] and heart rate [53]. Forgiveness may have positive health effects due to its ability to reduce both anger and stress [54] as well as its link with positive relationship quality [55]. Forgiveness, therefore, may serve as a critical resource for health in later life.

The influence of forgiveness on health may depend on transgression severity. Subjective ratings of transgression severity have been linked to forgiveness in multiple studies [48]. Specifically, transgressions perceived as more severe are associated with less forgiveness of these specific severe offenses [56, 57]. We build on this work in the current study to examine if transgression severity is linked with worse health and also to examine whether forgiveness has a moderating effect on the transgression severity-health link.

#### 1.4.1 Moderating Role of Forgiveness

In this study, we focus on the role of forgiveness as an interpersonal resource that may neutralize the experience of being hurt by a close social relationship later in life. Interpersonal resources can buffer the experience of stress on self-reported health status [58]. The ability to use an interpersonal resource such as forgiveness later in life may be particularly helpful in certain relationship contexts. For example, Allemand and colleagues [59] found a positive association between satisfaction with romantic relationships and forgiveness. Yet, it is not clear as to whether forgiveness serves as resource equally for older adults when the transgression occurs with a spouse/partner as opposed to another close relationship. McCullough’s [20, 21] valuable relationship hypothesis noted prior guides our expectation that forgiveness may play a stronger role in offsetting the negative influence on health of transgressions encountered in the marital/partner relationship compared to other close relationships. This is based on the presumed value placed on this relationship as well as increased chance for frequent contact. Among older adults, this value may in part be driven by the importance of the spouse/partner tie in helping to maintain health through management of health conditions and provision of essential caregiving support.

There is a paucity of research on forgiveness as a resource that may ameliorate the stress- health link. Though forgiveness has been cited as one stress coping strategy that may reduce the negative impact of stress on physical health [54, 60], it is unclear as to what type of forgiveness has positive effects on physical health in later life. For example, Toussaint and colleagues [60] found that being more forgiving generally (i.e., trait forgiveness) moderated the effect of lifetime stress severity on mental, but not physical health among a sample of college students. Toussaint and colleagues [60] note the need for more research to test the moderating role of forgiveness on the stress-physical health link at points beyond young adulthood and with other types of forgiveness (e.g., state forgiveness). State forgiveness may be especially important to examine in later life as this type of forgiveness accounts for the interpersonal context in which a specific transgression occurs.

### 1.5 Present Study

Considering the literature reviewed above, this study addresses three research questions:

1) Is there an association between transgression severity and older adults’ physical health? We hypothesize that older adults who report experiencing a more severe recent transgression committed by a close social relationship will report worse physical health.

2) Does state forgiveness (i.e., how likely to forgive a recent transgressor) moderate the association between transgression severity and physical health? We hypothesize generally that the interpersonal resource of forgiveness will help facilitate positive aging (i.e., better health) by helping older adults cope with and offset the negative impact of a recent transgression committed by someone close and important in their lives. More specifically, we hypothesize that among those reporting higher levels of state forgiveness, a more severe recent transgression will not be related to poor physical health. In contrast, among those reporting lower levels of state forgiveness, the negative association between transgression severity and physical health will still be present.

3) Does state forgiveness play a stronger role when the transgressor is a spouse/partner compared to another close social relationship? We hypothesize that among older adults reporting more state forgiveness, a transgression in the context of a marriage/partnership will be associated with better health compared to another close social relationship. Among older adults reporting less state forgiveness we hypothesize that transgressions experienced in the context of a marriage/partnership will be associated with worse health compared to another close social relationship.

## 2. Methods

### 2.1 Sample

Data are from the Detroit Community Survey, a cross-sectional survey of social relations, forgiveness, humility, and health in the Detroit Metropolitan Area [61, 62]. Surveys were conducted by trained interviewers of the Survey Research Center at the University of Michigan. Surveys were conducted via telephone during 2015–2016 using computer assisted interviewing and lasted on average 52 minutes. The overall cooperation rate among eligible households was 93%. For the present study, only data from respondents aged 50 and older were analyzed. The final sample of respondents with complete data on all study variables included 380 respondents. This study was approved by the University of Michigan Institutional Review Board (HUM#00099310) on March 4, 2015.

### 2.2 Measures

Physical health was measured as *self-rated health* using a single item “How would you rate your health at the present time?” Response options were reverse coded so that 1=poor and 5=excellent.

#### 2.2.1 Transgression Severity

Respondents were asked to “think about a recent time that you were seriously hurt, irritated, or annoyed.” Respondents who were married/living with a partner were asked to think about such a recent situation with their spouse/partner. Respondents who were not married/living with a partner were asked to think about a situation with one of their close social network members. Respondents were then asked how upset they were by the situation on a scale from 1 (not at all) to 5 (extremely).

#### 2.2.2 State Forgiveness

Following report of a recent transgression, respondents were asked how likely they would be to forgive the person who committed the recent transgression using the TRIM-12 scale *of state forgiveness* [63]. The TRIM-12 is the most widely used self-report measure of forgiveness [17]. This scale consists of 12 items ranging from strongly disagree (1) to strongly agree (5). Higher scores indicate a greater likelihood to forgive. Example items included, “I cut off the relationship with NAME” and “I withdrew from NAME”. State forgiveness was modeled as a latent variable in analyses.

Confirmatory factor analysis (CFA) was utilized to confirm the factor structure of the state forgiveness scale. Results of the CFA indicated that the model had adequate fit (χ^2^(35, N = 380) = 96.97, *p* < .001; CFI = .955; RMSEA = .068 (90% CI: .052, .085); SRMR = .044). Reliability of the scale using Cronbach’s alpha also revealed moderate-high reliability (α = .83).

#### 2.2.3 Martial / Partnership Status

Respondents were grouped into two categories, those who reported that they were currently married or living with a partner (1) and those who were not (0).

#### 2.2.4 Demographic Covariates

Age was measured by subtracting date of birth from interview date. Gender was coded as male (0) and female (1). Education was measured as the highest grade of school or year of college completed (0-17+). Race was measured with two dummy variables: Arab American (1) vs. White American (0) and African American (1) vs. White American (0). Religiosity (measured with a single item on a four-point scale ranging from not at all religious to very religious) was included during initial model testing as a covariate given prior research linking religiosity to forgiveness [64]. Religiosity was found not to be related to health in this sample, and no findings substantively changed when it was removed. Therefore, a decision was made not to include religiosity in the final models.

### 2.3 Analysis Plan

Structural equation modeling (SEM) was conducted primarily to allow for the modelling of state forgiveness as a latent variable. This analytic technique allows for the modeling of measurement error for latent constructs, which results in less biased parameter estimates. SEM also allowed for testing of our multiple hypothesized relationships between observed (non-latent) variables and the latent state forgiveness variable. First, we conducted a SEM model examining the direct relationship between transgression severity, state forgiveness, marital/partnership status and self-rated health, controlling for age, gender, race, and education. Next, we conducted a SEM model that included all main effects and added two interaction terms: transgression severity x state forgiveness and marital/partnership status x state forgiveness. In the interaction model, all continuous variables were mean centered to prevent multicollinearity.

Because typical model fit statistics for SEM models (CFI, RMSEA, SRMR) are not available in models that include an interaction involving a latent variable, model fit and change in model fit between the interaction model and the main effect model were determined using the −2 log likelihood difference chi-square test [65]. All analyses were conducted with Mplus 7.4 [66].

## 3. Results

Presented in Table 1 are sample characteristics and bivariate associations between all study variables. The age range for the overall sample was 50-94 years old with an average age of 63.7 (SD = 9.8). Sixty-six percent were female, and participants reported an average educational attainment of 14 years. Slightly more than half (53.7%) of the sample reported being currently married or living with a partner so therefore reported on the severity of a recent transgression committed by their spouse/partner. The rest of sample included 12.1% who were never married, 16.1% divorced, 2.9% separated, and 15.0% widowed. Those not married/living with a partner reported on a recent transgression committed by another (non-spouse/partner) close social relationship including: adult child (15.5%), sibling (11.6%), friend (6.8%), other family member (9.7%), other relationships (2.6%).

Average ratings of how severe the recent transgression committed by a spouse/partner or close relationship was 3.7 (SD=1.1; Range: 1-5). The average level of state forgiveness reported with the person who committed the transgression was 4.3 (SD=0.7; Range: 1-5). The sample had an average self-rated health score of between good and very good (M=3.6; SD=1.1; Range: 1-5).

### 3.1 Is There an Association between Transgression Severity and Older Adults’ Physical Health?

**Figure 1:**
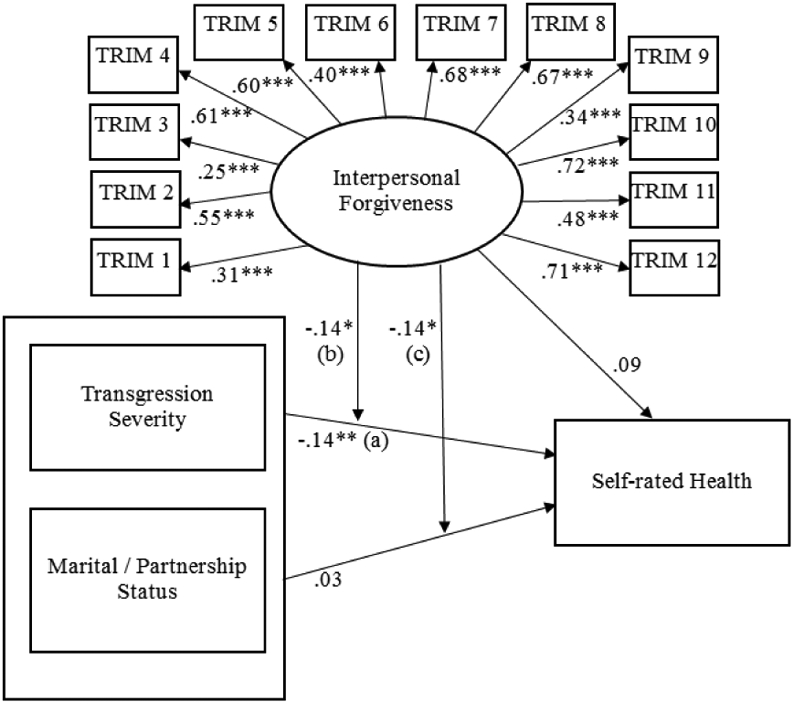
Standardized Results from Structural Equation Models. *p < .05; **p < .01; ***p < .001.

SEM was conducted to assess the direct link between severity of a recent transgression and self-rated health. Overall model results indicated the model was a good fit to the data, χ^2^(133, N = 380) = 330.501, *p* < .001; CFI = .87; RMSEA = .063 (90% CI: .054, .071); SRMR = .075. In support of our hypothesis, greater transgression severity was associated with worse self-rated health (*B* = −.13, *SE* = .05, *p* < .01 (see Table 2, Model 1 and Figure 1).

Next, to examine research questions 2 and 3, we conducted SEM to test moderation effects by adding interaction terms to model #1. Comparison of model fit between the direct effects model and the model with interaction terms revealed that the models were significantly different (−2 Log Likelihood = 9.64, *p* < .01). This indicates that inclusion of the interaction terms did provide a better explanation of relationships between the study variables.

### 3.2 Does State Forgiveness Moderate the Association between Transgression Severity and Physical Health?

**Figure 2:**
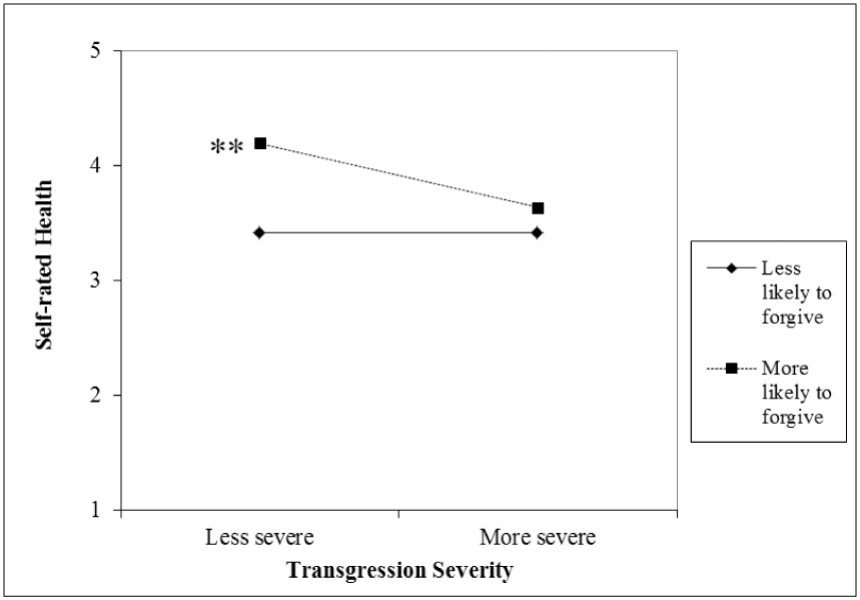
Association between transgression severity and self-rated health by state forgiveness. **p < .001.

The state forgiveness x transgression severity interaction was significantly associated with self-rated health (*B* = −.38, *SE* = .17, *p* < .05; see Table 2, Model 2). Details of the nature of the significant interaction and results from simple slopes analysis are presented in Figure 2.

Figure 2 shows that the association between transgression severity and self-rated health was significant among those who indicated they were more likely to forgive (*B* = −.25, SE = .07, *p* < .001). Specifically, we found that among older adults who were more likely to forgive their transgressor, experiencing a more severe transgression was associated with worse self-rated health. In contrast, among older adults who were less likely to forgive their transgressor, there was no association between transgression severity and self-rated health (*B* = −.00, SE = .09, *p* = .99). The nature of this interaction was contrary to the direction hypothesized, in that we expected having more of the resource of forgiveness would help to offset more severe transgressions. Instead it appears to only do so for less severe transgressions.

### 3.3 Does State Forgiveness Play a Stronger Role when the Transgressor is a Spouse/Partner Compared to Another Close Social Relationship?

**Figure 3:**
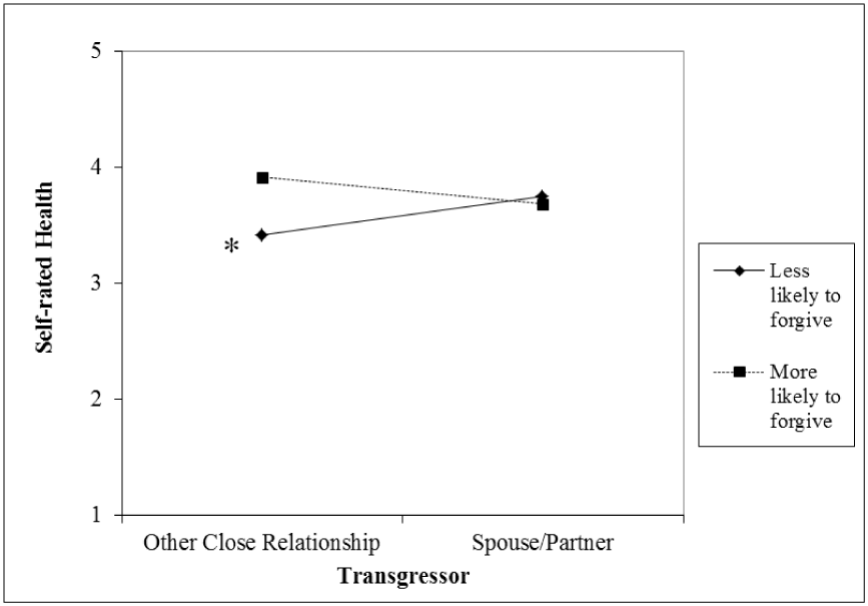
Association between transgressor relationship type and self-rated health by state forgiveness. *p < .05.

The state forgiveness x martial/partnership status interaction was significantly associated with self-rated health (*B* = −.84, SE = .38, *p* < .05; see Table 2, Model 2). Details of the nature of the significant interaction and results from simple slopes analysis are presented in Figure 3.

Figure 3 shows that among those reporting higher levels of state forgiveness (i.e., more likely to forgive their transgressor for the recent transgression), the association between who the transgressor was (i.e., spouse/partner versus another close social relationship) and self-rated health was not significant (*B* = −.23, SE = .17, p = .18). In contrast, among those reporting lower levels of state forgiveness, the transgressor being a close other social relationship was associated with worse health compared to when it was a spouse/partner (*B* = .33, SE = .17, *p* < .05. This result was contrary to our hypothesis in that more of the resource did not differentially have an impact across varying types of close transgressors.

## 4. Discussion

Given the strong link between close social relationships and positive aging, research is needed to better understand the implications of when these relationships are strained. Specifically, a better understanding of resources that can play a role in neutralizing hurtful interpersonal situations can help guide the development of positive aging interventions focused on close social relations. We found generally that experiencing a more severe transgression committed by a close social relationship including spouses/partners and other close ties was negatively associated with physical health. Further, state forgiveness was found to have a moderating role in this link. The interactive influence of this interpersonal resource operated in ways we did not expect, but, nevertheless, highlights its useful role in promoting positive aging and contributes to a growing understanding of how it operates. Specifically, this resource when available in greater amounts helps offset more minor events and occurrences (i.e., less severe transgressions). Further, and possibly more importantly, are the findings within the context of when state forgiveness is less available, i.e. when less likely to forgive a close social relationship when hurt by them. Specifically, when less available, both minor and more severe transgressions were found to have similarly negative influences on health. Additionally, when less available, transgressions committed within the context of other close relationships (not a marital/partner relationship) were associated with worse health.

### 4.1 Transgression Severity

Findings from this study are consistent with prior research and theory focused on transgressions (i.e., conflict, tensions and stress) experienced within the context of close social relationships. This study’s findings help to expand on previous empirical findings that not only the frequency of these situations [5], but also how severe they are, can impact physical health.

The finding that more severe transgressions were associated with reports of worse physical health provides further support for the stress-coping model of forgiveness. Specifically, this is due to interpersonal transgressions within the context of close social relationships operating in the same manner as stress has been demonstrated to affect health (i.e., greater stress associated with worse health). Various mechanisms through which interpersonal stress may impact physical health have been theorized in the stress-coping model of forgiveness [12, 13] and empirically documented including direct psychological [67] and physiological pathways [34, 67]. An interpersonal perspective suggests another potential explanation for this link is the essential role that close social relationships play in helping older adults manage health declines in later life. While, there is discussion in the forgiveness literature regarding how interpersonal stress can impact health indirectly through a negative impact on relationship quality [14], this process in later life specifically may be more complicated. It may be that strain or tension resulting from transgressions committed by one or more of these close ties could result in vulnerabilities in the management of complex health conditions. Therefore, when older adults are hurt by a close social relation they rely upon, it may erode trust and open-ness to receiving support in the future, both of which can lead to poor health.

### 4.2 Moderating Role of State Forgiveness

#### 4.2.1 Transgression Severity

Our hypothesis regarding the role of state forgiveness in helping to neutralize the impact of more severe transgressions in the context of close social relationships was not supported. However, our findings do shed light on when this resource is most beneficial as well as when it is less available the implications for the stress-health link. We found that in the context of having greater availability of this interpersonal resource, less severe transgressions are associated with better physical health. This suggests there may be a point of diminishing returns in terms of the ability of state forgiveness to offset the negative impact of transgressions on health. In contrast, when there is less availability of this interpersonal resource, both more and less severe transgressions have near equal negative influences on physical health.

These findings contribute to a growing understanding of how diverse types of forgiveness moderate the stress-health link. For example, Toussaint and colleagues [60] found that trait forgiveness helps to offset the negative effect of stress on mental health, but not physical health. This finding and findings from the present study when considered together suggest trait forgiveness can influence the stress-mental health link while state forgiveness may be needed to offset the stress-physical health link. However, Toussaint and colleagues’ study was conducted among college aged students and the present study among adults age 50 and older. This suggests further research is needed to examine and compare the moderating effects of both trait and state forgiveness on the stress-health link across the life span.

Findings from the present study show the strength of state forgiveness to limit the impact of less severe transgressions, which may be more common and frequent. Therefore, state forgiveness plays an essential role in promoting positive aging in this context. Given the observed diminishing returns of state forgiveness for coping with more severe transgressions, further research is needed to determine resources that can help older adults manage and cope with these more severe transgressions committed by someone close to them. One such possibility is the use of behavior focused coping strategies related to forgiveness, described by Worthington [14] as decisional forgiveness. While argued to not likely alter the stress-health link directly [14], this coping strategy may help repair damaged relationships which may result in better health in the longer term.

#### 4.2.2 Relationship Context

We also found that state forgiveness significantly moderated the association between who the transgressor was and physical health. This finding generally confirms the important role of relationship context described in both the interpersonal process of forgiveness and unforgiveness [11], and the stress-coping model of forgiveness [12, 13]. Specifically, we found that when state forgiveness was less available, transgressions committed within the context of other close relationships were associated with worse health compared to a marriage/partnership context.

One possible explanation for this finding is that transgressions committed within a marriage/partnership may become more normative over time. Therefore, when forgiveness is less available there is no negative impact on health. This is consistent with Birditt and colleagues’ [26] study of longitudinal patterns of negative social relations in which they found that negative relationship quality increases over time among spouses/partners [26]. This could be a function of close proximity (i.e., greater likelihood of living in same household) and frequency of interactions which may increase chances of transgressions occurring. Similarly, in another study, Birditt and colleagues [68] found that older compared to younger adults were more likely to report tensions with a spouse/partner, less likely to argue, and less likely to respond to tensions.

Another possible explanation for this finding is that transgressions experienced in the spousal/partner relationship may not rise to the level of harming the tie to the point where health is impacted. This is supported in the data examined in this study as only 19.7% of those reporting on a recent transgression committed by a spouse/partner indicated it was a severe transgression (i.e., they were extremely upset). In contrast, 39.0% of respondents reporting on a transgression committed by another close social relationship reported that it was a severe transgression. A third potential explanation for this finding is that these non-spousal/partner ties do not have as strong of a foundation or history of positive interactions and support exchanges.

All three of these potential explanations are consistent with McCullough’s [20, 21] valuable relationship hypothesis. These other close relationships may be valued differently compared to the spousal/partner relationship. As a result, there may be different motivation levels to return to pre-transgression levels of interaction. Therefore, when state forgiveness is less available, transgressions committed by other close social relationships may be more detrimental to health. In the context of transgressions committed by a spouse/partner, the transgressions appear to be more minor (e.g., annoyances) and thus forgiveness may not be warranted or needed. Further, the increased opportunity for contact (if the spouse/partner are living together) may increase motivation to return to pre-conflict interaction thus reducing the potential for impacting health.

One mechanism through which state forgiveness may help to promote health when hurt by a non-spouse partner is through protection or maintenance of a diverse set of ties. Therefore, when state forgiveness in this context is lacking, transgressions may go unresolved and potentially lead to social isolation [36]. Staying socially engaged and having a diverse social network or many types of social ties later in life is essential for maintaining good health [9, 69, 70]. Being able to forgive in the context of these relationships may help ensure diversity of ties and prevent social isolation.

### 4.3 Practice Implications

Although some are contrary to hypothesized directions, results from this study provide important and relevant information for clinical practice. In particular. findings from this study can be helpful for counselors seeking therapy alternatives for older adults, and spiritual and religious leaders providing pastoral care to older congregational members. The following findings may help to inform practice: 1) Emotional state forgiveness as a coping mechanism can be equally beneficial when the transgressor is a spouse/partner or other close social relationship. 2) When less likely to forgive, transgressions both big and small can have harmful effects on health. 3) Additional types of forgiveness strategies, e.g., decisional forgiveness, may be needed to help reduce the negative health impact of more severe transgressions on health. Future research should examine whether such alternatives to emotional forgiveness are effective.

### 4.4 Limitations and Future Directions

This study has a number of limitations. First, we were not able to disentangle the impact of being married/living with partner vs. not on health from experiencing a transgression committed by a spouse/partner. This was due to our research design. People who were married/living with a partner were only asked to think about and report on a recent hurtful situation with a spouse/partner. Respondents did not have the option of reporting on such a situation with another close social relationship. Future studies should ask people who are married or living with a partner to report on the experience of transgressions committed both by their spouse/partner as well as other close social relationships. Such data will allow for a comparison among those who are married/living with a partner of the differential impact of experiencing transgressions committed by a spouse/partner as well as other close social relationships on health.

A second limitation is that we only investigated the unique effect of transgression severity on health. Future studies with larger samples can examine two-way interactions between who specifically committed the transgression (e.g., adult child, sibling, friend) and transgression severity. Doing so will allow for examination of whether transgressions that are more severe within the context of specific relationships are more or less harmful to health compared to other relationships. Such information can help to further tailor interventions to specific relationships and situations.

A third limitation relates to our use of the TRIM-12 to measure state forgiveness. This scale was designed to measure motivations toward unforgiveness, which has been conceptualized as related to but unique form forgiveness [13]. Future studies examining state forgiveness in the context of a recent transgression can address this limitation by using the more recently developed TRIM-18 which includes a benevolence subscale [21].

A fourth limitation of this study is that we did not investigate how key personal characteristics may moderate the processes examined in this study. Future research is needed to better understand how characteristics of the individual (e.g., age, gender, race/ethnicity) may serve as additional important contexts that influence the links between forgiveness, interpersonal transgressions, and health.

## 5. Conclusions

This study contributes to a growing literature, which highlights forgiveness as a resource that older adults can draw upon to help manage the experience of transgressions. In particular, this study helps to provide better understanding of how interpersonal stress, specifically experiencing transgressions within the context of close social relationships can be harmful to older adults. Forgiving in the context of close relationships may be beneficial not only for the individual themselves, but also for the relationship, which can have important and unique health implications in later life. Overall, findings highlight the importance and potentially broad impact of forgiveness as a resource to facilitate positive aging.

## Figures and Tables

**Table 1:** Sample characteristics and correlations.

Measure	1	2	3	4	5	6	7	8	9
1. Age	1.00								
2. Female	−.05	1.00							
3. Arab American	− 22**	−.06	1.00						
4. African American	−.08	.18**	−.34**	1.00					
5. Education	−.07	−.13**	.06	−.17**	1.00				
6. Married/Living with partner	−.19**	−.15**	.26**	−.29**	.12*	1.00			
7. Transgression severity	−.13*	.13*	−.03	.17**	−.01	−.20**	1.00		
8. State forgiveness	.12*	−.06	.03	−.09	−.01	.15**	μ.35**	1.00	
9. Self-rated health	.02	−.12*	.06	−.16**	.19**	.13*	−.20**	.16**	1.00
Mean / %	63.7	65.8	14.2	40.5	14.1	53.7	3.7	4.4	2.3
SD	9.8				2.2		1.1	0.6	0.9

* *p* < .05;

** *p* < .01

**Table 2:** Structural equation models examining the effects of characteristics of a recent transgression on self-rated health.

Parameter	Model 1			Model 2		
	B	SE	β	B	SE	β
Age	.00	.01	−.00	−.00	.01	−.01
Female	−.13	.11	−.06	−.13	.11	−.06
Arab American v. White American	.01	.16	.00	.00	.16	.00
African American v. White American	−.18	.12	−.09	−.17	.12	−.08
Education	.08**	.02	.16	08**	.02	.16
Spouse/Partner Transgressor	.07	.11	.03	.05	.11	.02
Transgression severity	−.13**	.05	−.14	−.13*	.05	−.14
State forgiveness	.28	.19	.09	.74**	.27	.24
State forgiveness x Spouse/Partner transgressor				−.84*	.38	−.14
State forgiveness x transgression severity				−.38*	.17	−.14
−2 Log Likelihood	13226.70			13217.06		
−2 Log Likelihood					9.64**	
R^2^		.09			.11	

* *p* < .05;

** *p* < .01;

*** *p* < .001

## References

[1] Ranzijn R The potential of older adults to enhance community quality of life: Links between positive psychology and productive aging. Ageing Int. 2002; 27: 30–55.

[2] Peterson C, Seligman MEP. Character strengths and virtues: A handbook and classification. New York, N.Y: Oxford University Press; 2004. 815 p.

[3] McCullough ME, Witvliet CV. The psychology of forgiveness. Snyder C, Lopez S, editors. Handb Posit Psychol. 2002; 2: 446–458.

[4] Fincham FD. Forgiveness: Integral to a science of close relationships? In 2009 [cited 2018 Dec 1]. Available from: http://www.academia.edu/2870624/Forgiveness_Integral_to_a_science_of_close_relationships

[5] Laursen B, Hafen CA. Future directions in the study of close relationships: Conflict is bad (except when it’s not). Soc Dev. 2010; 19: 858–872. 20953335 10.1111/j.1467-9507.2009.00546.x PMC2953261

[6] Rook KS. Social networks in later life: Weighing positive and negative effects on health and well-being. Curr Dir Psychol Sci. 2015; 24: 45–51. 26366047 10.1177/0963721414551364 PMC4564251

[7] Rowe J, Kahn R. Human aging: Usual and successful. Science. 1987; 237: 143–149. 3299702 10.1126/science.3299702

[8] Rowe JW, Kahn RL. Successful Aging. Gerontologist. 1997; 37: 433–440. 9279031 10.1093/geront/37.4.433

[9] Rowe JW, Kahn RL. Successful aging 2.0: Conceptual expansions for the 21st century. J Gerontol B Psychol Sci Soc Sci. 2015; 70: 593–596. 25878054 10.1093/geronb/gbv025

[10] Seligman MEP. Flourish. New York, N.Y: Free Press; 2011.

[11] Worthington EL Jr, Wade NG. The psychology of unforgiveness and forgiveness and implications for clinical practice. J Soc Clin Psychol. 1999; 18: 385–418.

[12] Strelan P, Covic T. A review of forgiveness process models and a coping framework to guide future research. J Soc Clin Psychol. 2006; 25: 1059–1085.

[13] Worthington EL Jr. Forgiveness and reconciliation: Theory and application. New York, N.Y: Brunner-Routledge; 2006.

[14] Worthington EL Jr, Witvliet CVO, Pietrini P, Miller AJ. Forgiveness, health and well-being: A review of evidence for emotional versus decisional forgiveness, dispositional forgiveness, and reduced unforgiveness. J Behav Med. 2007; 30: 291–302. 17453329 10.1007/s10865-007-9105-8

[15] Lazarus RS, Folkman S. Stress, appraisal, and coping. New York, N.Y: Springer publishing company; 1984.

[16] Lazarus R A new synthesis: Stress and emotion. New York, N.Y: Springer publishing company; 1999.

[17] Worthington EL, Lavelock C, vanOyen Witvliet C, Rye MS, Tsang JA, Toussaint L. Measures of forgiveness: Self-report, physiological, chemical, and behavioral indicators. In: Boyle GJ, Saklofske DH, Matthews G, editors. Measures of personality and social psychological constructs. San Diego: Academic Press; 2015. p. 474–502.

[18] McCullough ME, Pargament Kl, Thoresen CE. Forgiveness: Theory, research, and practice. New York, N.Y: Guilford Press; 2000.

[19] Paleari FG, Regalia C, Fincham FD. Measuring offence-specific forgiveness in marriage: The Marital Offence-Specific Forgiveness Scale (MOFS). Psychol Assess. 2009; 21: 194–209. 19485674 10.1037/a0016068

[20] McCullough M Beyond revenge: The evolution of the forgiveness instinct. San Francisco: John Wiley & Sons; 2008.

[21] McCullough ME, Luna LR, Berry JW, Tabak BA, Bono G. On the form and function of forgiving: Modeling the time-forgiveness relationship and testing the valuable relationships hypothesis. Emotion. 2010;10: 358–376. 20515225 10.1037/a0019349

[22] Antonucci T, Akiyama H, Takahashi K. Attachment and close relationships across the life span. Attach Hum Dev. 2004; 6: 353–370. 15764124 10.1080/1461673042000303136

[23] Lang FR, Carstensen LL. Close emotional relationships in late life: further support for proactive aging in the social domain. Psychol Aging. 1994; 9: 315. 8054179 10.1037//0882-7974.9.2.315

[24] Holt-Lunstad J The potential public health relevance of social isolation and loneliness: Prevalence, epidemiology, and risk factors. Public Policy Aging Rep. 2017; 27: 127–130.

[25] Antonucci TC, Akiyama H. Stress and coping in the elderly. Appl Prev Psychol. 1993; 2: 201–208.

[26] Birditt KS, Jackey LM, Antonucci TC. Longitudinal patterns of negative relationship quality across adulthood. J Gerontol B Psychol Sci Soc Sci. 2009; 64: 55–64. 19228921 10.1093/geronb/gbn031 PMC2654992

[27] Rook KS. The negative side of social interaction: Impact on psychological well-being. J Pers Soc Psychol. 1984; 46: 1097–1108. 6737206 10.1037//0022-3514.46.5.1097

[28] Villalonga-Olives E, Kawachi I. The dark side of social capital: A systematic review of the negative health effects of social capital. Soc Sci Med. 2017; 194: 105–127. 29100136 10.1016/j.socscimed.2017.10.020

[29] Tosevski DL, Milovancevic MP. Stressful life events and physical health. Curr Opin Psychiatry. 2006; 19: 184–189. 16612201 10.1097/01.yco.0000214346.44625.57

[30] Farrell AK, Simpson JA. Effects of relationship functioning on the biological experience of stress and physical health. Curr Opin Psychol. 2017; 13: 49–53. 28813293 10.1016/j.copsyc.2016.04.014

[31] Schneiderman N, Ironson G, Siegel SD. Stress and health: Psychological, behavioral, and biological determinants. Annu Rev Clin Psychol. 2005; 1: 607–628. 17716101 10.1146/annurev.clinpsy.1.102803.144141 PMC2568977

[32] Bolger N, DeLongis A, Kessler RC, Schilling EA. Effects of daily stress on negative mood. J Pers Soc Psychol. 1989; 57: 808. 2810026 10.1037//0022-3514.57.5.808

[33] Sheets ES, Craighead WE. Comparing chronic interpersonal and noninterpersonal stress domains as predictors of depression recurrence in emerging adults. Behav Res Ther. 2014; 63: 36–42. 25277497 10.1016/j.brat.2014.09.001 PMC4258528

[34] Kiecolt-Glaser JK, Gouin JP, Hantsoo L. Close relationships, inflammation, and health. Neurosci Biobehav Rev. 2010; 35: 33–38. 19751761 10.1016/j.neubiorev.2009.09.003 PMC2891342

[35] Buerki S, Adler RH. Negative affect states and cardiovascular disorders: A review and the proposal of a unifying biopsychosocial concept. Gen Hosp Psychiatry. 2005; 27: 180–188. 15882764 10.1016/j.genhosppsych.2004.12.003

[36] Cohen S Social relationships and health. Am Psychol. 2004; 59: 676–684. 15554821 10.1037/0003-066X.59.8.676

[37] Cacioppo JT, Hawkley LC. Social isolation and health, with an emphasis on underlying mechanisms. Perspect Biol Med. 2003; 46: S39–S52. 14563073

[38] Courtin E, Knapp M. Social isolation, loneliness and health in old age: A scoping review. Health Soc Care Community. 2017; 25: 799–812. 26712585 10.1111/hsc.12311

[39] Rook KS. Emotional health and positive versus negative social exchanges: A daily diary analysis. Appl Dev Sci. 2001; 5: 86–97.

[40] House JS, Landis KR, Umberson D. Social relationships and health. Science. 1988; 241: 540–545. 3399889 10.1126/science.3399889

[41] Lillard LA, Waite LJ. ‘Til death do us part: Marital disruption and mortality. Am J Sociol. 1995; 100: 1131–1156.

[42] Waite L, Gallagher M. The case for marriage: Why married people are happier, healthier, and better off financially. New York, N.Y: Broadway Books; 2001.

[43] Antonucci TC, Lansford JE, Akiyama H. Impact of positive and negative aspects of marital relationships and friendships on well-being of older adults. Appl Dev Sci. 2001; 5: 68–75.

[44] Kiecolt-Glaser JK, Loving TJ, Stowell JR, Malarkey WB, Lemeshow S, Dickinson SL, Hostile marital interactions, proinflammatory cytokine production, and wound healing. Arch Gen Psychiatry. 2005; 62: 1377–1384. 16330726 10.1001/archpsyc.62.12.1377

[45] Birditt KS, Newton NJ, Cranford JA, Webster NJ. Chronic stress and negative marital quality among older couples: Associations with waist circumference. J Gerontol Ser B. 2019; 74: 318–328. 10.1093/geronb/gbw112 PMC6327652 27664418

[46] Toussaint LL, Williams DR, Musick MA, Everson SA. Forgiveness and health: Age differences in a US probability sample. J Adult Dev. 2001; 8: 249–257.

[47] Enright RD, Santos MJ, Al-Mabuk R. The adolescent as forgiver. J Adolesc. 1989; 12: 95–110. 2708604 10.1016/0140-1971(89)90092-4

[48] Riek BM, Mania EW. The antecedents and consequences of interpersonal forgiveness: A meta-analytic review. Pers Relatsh. 2012; 19: 304–325.

[49] Toussaint LL, Owen AD, Cheadle A. Forgive to live: Forgiveness, health, and longevity. J Behav Med. 2012; 35: 375–386. 21706213 10.1007/s10865-011-9362-4

[50] Lawler KA, Younger JW, Piferi RL, Billington E, Jobe R, Edmondson K, A change of heart: Cardiovascular correlates of forgiveness in response to interpersonal conflict. J Behav Med. 2003; 26: 373–393. 14593849 10.1023/a:1025771716686

[51] Wilson T, Milosevic A, Carroll M, Hart K, Hibbard S. Physical health status in relation to self-forgiveness and other-forgiveness in healthy college students. J Health Psychol. 2008; 13: 798–803. 18697892 10.1177/1359105308093863

[52] Friedberg JP, Suchday S, Shelov DV. The impact of forgiveness on cardiovascular reactivity and recovery. Int J Psychophysiol. 2007; 65: 87–94. 17466400 10.1016/j.ijpsycho.2007.03.006

[53] C van O Witvliet, Ludwig TE, Laan KLV. Granting forgiveness or harboring grudges: Implications for emotion, physiology, and health. Psychol Sci. 2001; 12: 117–123. 11340919 10.1111/1467-9280.00320

[54] Worthington EL Jr, Scherer M. Forgiveness is an emotion-focused coping strategy that can reduce health risks and promote health resilience: Theory, review, and hypothesis. Psychol Health. 2004; 19: 385–405.

[55] Toussaint L, Jorgensen KM. Inter-parental conflict, parent-child relationship quality, and adjustment in Christian adolescents: Forgiveness as a mediating variable. J Pschology Christ. 2008; 27: 337–346.

[56] Wade NG, Worthington EL Jr. Overcoming interpersonal offenses: Is forgiveness the only way to deal with unforgiveness? J Couns Dev. 2003; 81: 343–353.

[57] Fincham FD, Jackson H, Beach SR. Transgression severity and forgiveness: Different moderators for objective and subjective severity. J Soc Clin Psychol. 2005; 24: 860–875.

[58] Cohen CI, Teresi J, Holmes D. Social networks, stress, and physical health: A longitudinal study of an inner-city elderly population. J Gerontol. 1985; 40: 478–486. 4008882 10.1093/geronj/40.4.478

[59] Allemand M, Amberg I, Zimprich D, Fincham FD. The role of trait forgiveness and relationship satisfaction in episodic forgiveness. J Soc Clin Psychol. 2007; 26: 199–217.

[60] Toussaint L, Shields GS, Dorn G, Slavich GM. Effects of lifetime stress exposure on mental and physical health in young adulthood: How stress degrades and forgiveness protects health. J Health Psychol. 2016; 21: 1004–1014. 25139892 10.1177/1359105314544132 PMC4363296

[61] Antonucci TC, Ajrouch KJ, Webster NJ, Birditt KS. Social networks and forgiveness: The role of trust and efficacy. Res Hum Dev. 2018; 15: 3–20. 10.1080/15427609.2017.1414670 PMC6377239 30774568

[62] Webster NJ, Ajrouch KJ, Antonucci TC. Sociodemographic differences in humility: The role of social relations. Res Hum Dev. 2018; 15: 50–71. 30774568 10.1080/15427609.2017.1414670 PMC6377239

[63] McCullough ME, Rachal KC, Sandage SJ, Worthington EL Jr, Brown SW, Hight TL. Interpersonal forgiving in close relationships: II. Theoretical elaboration and measurement. J Pers Soc Psychol. 1998; 75: 1586–1603. 9914668 10.1037//0022-3514.75.6.1586

[64] Krause N. Compassion, acts of contrition, and forgiveness in middle and late life. Pastor Psychol. 2016; 65: 127–141.

[65] Klein A, Moosbrugger H. Maximum likelihood estimation of latent interaction effects with the LMS method. Pschyometrika. 2000; 65: 457–474.

[66] Muthén L, Muthén B. MPlus. Los Angeles, CA: Muthén & Muthén; 2015.

[67] Bolger N, DeLongis A, Kessler RC, Schilling EA. Effects of daily stress on negative mood. J Pers Soc Psychol. 1989; 57: 808. 2810026 10.1037//0022-3514.57.5.808

[68] Birditt K, Fingerman K, Almeida D. Age differences in exposure and reactions to interpersonal tensions: A daily diary study. Psychol Aging. 2005; 20: 330–340. 16029096 10.1037/0882-7974.20.2.330

[69] Fiori K, Antonucci T, Akiyama H. Profiles of social relations among older adults: A cross-cultural approach. Ageing Soc. 2008; 28: 203–231.

[70] Webster N, Antonucci T, Ajrouch K, Abdulrahim S. Social networks and health among older adults in Lebanon: The mediating role of support and trust. J Gerontol B Psychol Sci Soc Sci. 2014; 70: 155–166. 25324295 10.1093/geronb/gbu149 PMC4861646

